# *Angiostrongylus cantonensis* Infection on Mayotte Island, Indian Ocean, 2007-2012

**DOI:** 10.1371/journal.pntd.0004635

**Published:** 2016-05-04

**Authors:** Loïc Epelboin, Renaud Blondé, Abdourahim Chamouine, Alexandra Chrisment, Laure Diancourt, Nicolas Villemant, Agnès Atale, Claire Cadix, Valérie Caro, Denis Malvy, Louis Collet

**Affiliations:** 1 Department of Prevention, Actions and Public Health, Centre Hospitalier de Mayotte, Mamoudzou, Mayotte; 2 Infectious and Tropical Diseases Department, Centre Hospitalier Andrée Rosemon, Cayenne, French Guiana; 3 Intensive Care Unit, Centre Hospitalier de Mayotte, Mamoudzou, Mayotte; 4 Department of Pediatrics, Centre Hospitalier de Mayotte, Mamoudzou, Mayotte; 5 Institut Pasteur, Environment and Infectious Risks Research and Expertise Unit, Paris, France; 6 Geography Institute (UFR08)—Paris 1 Panthéon-Sorbonne University, Paris, France; 7 Medical Biology Laboratory, Centre Hospitalier de Mayotte, Mamoudzou, Mayotte; 8 Victor Segalen University, Bordeaux 2, Bordeaux, France; University of Queensland, AUSTRALIA

## Abstract

**Introduction:**

Human angiostrongyliasis (HA) is a neurological helminthic disease caused by the lung worm *Angiostrongylus cantonensis*. It is suspected in the combination of travel or a residence in an endemic area and eosinophilic meningitis. In Mayotte, an island in the Indian Ocean, cases are rare but regular. The main objective of our study was to describe the epidemiological and diagnosis clues of HA in Mayotte. The secondary objectives were to evaluate the contribution of Real-Time Polymerase Chain Reaction (RT- PCR) for the diagnosis of HA, delineate the characteristics of the local transmission and ascertain the presence of *A*. *cantonensis* in *Achatina fulica*, the potential vector of the disease.

**Materials and Methods:**

Between 2007 and 2012, all cases of eosinophilic meningitis were retrospectively included and investigated by RT- PCR in the CSF. Descriptive analysis was conducted for clinical, biological and radiological features, and were analyzed for all patients together with the search for prognostic factors for mortality. Concurrently, geolocalization and temporal parameters were studied to correlate the occurrence of the cases with rainfall seasons and snails were collected to enhance a parasitic carriage with real time PCR.

**Results:**

During the 6-year period of the study, 14 cases were identified (2.3 cases/year) and 9 among 10 remaining CSF were positive in PCR. Among 14 cases of EM, 13 were less than 2 year-old children. The 1 year mortality rate was 5/14 (35.7%). Among survivors, 3/7 (42.8%) presented neurological sequelae. Factors associated with mortality were dysfunction of cranial nerves, abnormal brain imaging, and CSF glucose level inferior to 2 mmol/l. Occurrence of cases was temporarily and spatially correlated to the rainy season. Among the 64 collected giant snails, 6 (9.4%) were positive with *A*. *cantonensis* PCR. The likely main route of transmission was the children licking snails, carriers of the parasite.

**Conclusion:**

In Mayotte, HA was mainly found in paediatric cases under 2 years old, and evidenced a life-threatening disease. PCR seems to be a promising tool in the definitive diagnosis of HA. Population should be aware of the role of *A*. *fulica*, and not let the children have direct contact with the snails.

## Author Summary

Human angiostrongyliasis is a neurological helminthic disease caused by the lung worm *Angiostrongylus cantonensis*, and most cases are reported from Asia, particularly Thailand and China. In Mayotte, an overseas French Territory in the Indian Ocean, cases are rare but regular, with life-threatening clinical pictures in very young children, though the medical literature describes this disease as mild, even in children. We aimed to study this disease in the specific context of the Indian Ocean. We found that, although this disease is quite rare: 14 cases in a study period of 6 years, with evidence among children, and mainly (13/14) those less than 2 years old, and its prognosis is poor as the 1-year mortality rate was 35.7%, and neurological sequelae 42.7% of the survivors. The real time PCR performed on the CSF was a sensitive tool. The occurrence of cases was temporarily and geographically linked to rain, and 9.4% of the collected *Achatina fulica*, the giant African snails, were positive for the parasite with high parasitic loads. The specific epidemiology is linked to the bad habit of the young children to lick the snails, which are present everywhere on the island, and have a severe presentation due to the high parasitic load.

## Introduction

Human angiostrongyliasis (HA), also called nervous angiostrongyliasis, is a parasitic disease due to the lungworm *A*. *cantonensis*. *A*. *cantonensis* has been described for the first time in a rat in China, and the first human case has been reported in Taiwan in 1945 [1, 2]. The disease has been described progressively worldwide but is mainly endemic in China, South-eastern Asia and in the Pacific Ocean islands where outbreaks or sporadic cases have been reported [3]. Elsewhere cases are generally reported in travelers returning from endemic areas and sporadic autochthonous cases such as in the Pacific Islands [4], Brazil [5] and Jamaica [6]. The disease is known in the Indian Ocean area as well and *A*. *cantonensis* has been found in the snail and the rat on La Reunion Island, Maurice and Madagascar [7, 8]. Furthermore, several non confirmed human cases have been reported since 1977 on La Reunion Island [9, 10]. On Mayotte Island, the disease was first reported in 1996 in a 11-month old child [11]. Eight supplementary probable cases have reported until 2006, among which 5 children and 3 adults, all with a critical outcome [12, 13].

The sources of transmission vary according to the geographical area, but are generally related to the consumption of raw or undercooked intermediate host such as slugs and snails or paratenic host referring to shrimps or crabs [14]. Some clusters have also been linked to raw vegetables contaminated with slug or snail slime [15]. Following transmission, HA pathogenesis refers to an aberrant route with a tropism to the central nervous system.

Thus, the most common presentation of HA is an eosinophilic meningitis frequently accompanied with encephalitis signs, epilepsy and cranial nerves disorders [14]. The disease generally occurs in adults, due to the food-linked source of contamination, although pediatric cases have been reported [16]. Most cases are described to be mild and self-limited, even in children, although some fatal issues have been evidenced [3, 14]. We aimed to study the specificities of the local parasitic life cycle. For this purpose, we used Real time PCR (RT-PCR) for diagnosis of human *A*. *cantonensis* infection a new tool published in 2010 [17].

We aimed to 1) evaluate the incidence of EM between 2007 and 2012 (suspected cases of HA), 2) describe their individual clinical, biological, imaging characteristics, 3) determinate the variables associated to mortality, 4) evaluate the performance of *A*. *cantonensis* PCR in CSF for accurate diagnosis of HA, 5) ascertain the contextual and environmental variables, and 6) conduct an ancillary entomological analysis with a the use of the PCR to enhance the carriage of the parasite by *Achatina fulica*.

## Methods

### Setting

Mayotte is a French island of the Comoros archipelago, in the South-West Indian Ocean, where 217.000 inhabitants lived in 2012 and 50% of the population is under 17.5 years old (source: http://www.insee.fr). The island enjoys a tropical maritime climate. There are two seasons with a hot and wet rainy season flowing in from November to April with abundant precipitations and a dry season from May to October. In Mayotte, the vectors of transmission remain unclear, although the African giant snail, *A*. *fulica*, has been incriminated, like in the other places of Indian Ocean.

### Study design and participants

A cross-sectional study was conducted in the hospital of Mamoudzou, which is the main hospital of the island, and where all severe inpatients refer. EM is systematically followed-up among patients with CSF analysis since 2007. All the patients admitted for EM in any department of the hospital from January 2007 to December 2012 were thus longitudinally identified.

### Case definition and inclusion and exclusion criteria

As usually admitted in the literature, we define presumptive cases for patients who had clinical and biological criteria corresponding to the diagnosis of HA, probable cases corresponded to those who had a positive serology, and confirmed cases were those whom *A*. *cantonensis* was isolated in the CSF, by direct microscopy or with PCR. Clinical and biological criteria were based on the association of any neurological symptom with EM, defined by the presence of more than 10 eosinophil per millimeter cubic in the cerebrospinal fluid (CSF), or ≥ 10% of the total CSF leukocyte count. Patients with a false eosinophilia in the CSF due to a traumatic lumbar puncture, with blood eosinophilia from another etiology were excluded.

### Variables, data collection and analysis

The following variables were collected: epidemiological data (age, gender, place of birth, place to live), risk factor for transmission with a Shimaore (Comorian language of Mayotte) translator, such as the knowledge of the contact with a mollusk, medical history, clinical presentation, biological results (including blood and CSF), imaging features (brain scan or Magnetic Resonance Imaging (MRI)), treatments, date of last contact, and outcome at 1 month and 1 year after discharge (referred as dead, alive, with neurological sequelae or healthy).

The median and interquartile ranges were used for most of the continuous variables. Some of biological variables were categorized following the laboratory cut-off values or using the median and they were dichotomized because of the small sample size. Association between variables and 1-year mortality was obtained comparing alive and deceased patients’ variables with Fisher’s exact test for categorical variables and with Mann-Whitney Test for continual variables. All these data were anonymized in a standardized case report form and entered in the database. Data were analyzed with Stata IC 12.0, version 2.15.3.

### Ethic statement

The variables were anonymously and retrospectively collected in the medical charts. As far as ethical considerations are taken into account, the French National Commission on Informatics and Liberties authorizes the retrospective use of anonymous patient files on the site of patient care in a single hospital.

### Contextual variables linked to transmission

#### Environmental factors

The link between case reports and environmental factors, i.e. seasonality, pluviometry and presence of an infected vector were systematically evaluated. Thus, geolocalization of cases’ place of residence on the island was generated and correlated to pluviometry data on this behalf of pluviometry data of the 12 meteorological stations of Mayotte from 2007 to 2013 were obtained (source: Météo-France). These data were summarized to obtain a global monthly precipitations level on the island. Herein, the curve for cases incidence was analyzed for patterns of consistency with pluviometry data.

#### Malacological investigations

In every place or village where a case had been identified, African giant snails, *A*. *fulica*, were collected for analysis by real time PCR [17]. Five snails were collected in every place, and additional snails were collected in certain places where a positive result was found.

### Biological analysis

#### Serodiagnosis

From 2007 to 2010, samples provided for serological testing were sent to the only lab realizing an angiostrongyliasis infection diagnosis on the French territory, overseas regions included (laboratory of Centre Hospitalier de Gonesse, F-95500 Gonesse, France. They were performing an home-made method by the detection of specific antibodies against *A*. *cantonensis* antigens using an indirect immunofluorescence assay. The worms were inserted into a young and healthy rat heart to form a sort of "roulade". After freezing, cuts were made approximately 3 microns with a microtome (cryocut) and were attached to microscope slides to perform indirect immunofluorescence assay. The sera were diluted in two to two dilution to 1/40. Samples were introduced pure and diluted two in two. A conjugated antibody (total immunoglobulins) marked with fluorescein was used. Due to logistical constraints and specific reagents availability, patients’ serodiagnosis was stopped in 2010 in the reference laboratory, and no other lab was then performing this test anymore in France.

#### *Angiostrongylus cantonensis* real time PCR

Real time PCR realization: PCR was retrospectively performed in December 2013 and January 2014 among the remaining frozen CSF of patients with probable angiostrongyliasis, as well as on 10 control CSF samples: 5 with meningitis diagnosis (enterovirus = 1; *Klebsiella pneumoniae* = 1, *Haemophilus influenzae* = 1, *Streptococcus pneumoniae* = 2) and 5 CSF negative for herpes simplex virus 1&2 PCR. The biological method used was inspired from the publication of Qvarnstrom et al. (16). DNA preparation: Total DNA from human LCR (500 μl) was prepared using MagNA Pure compact nucleic acid isolation Kit Large volume (Roche Diagnostics) with a 50 μl elution volume. Concerning the analysis of the snails, 50 mg of different parts of the mollusk (midsection, tail, back, head) is added with 1300 μl of MagNA Pure 96 bacteria lysis buffer (Roche Diagnostics) an 200 μl of Proteinase K (Roche Diagnostics) and incubate at +65°C until the sample is completely disintegrated and after incubate 10 min at +95°C.500 μl of this sample is extracted like human LCR. PCR conditions: The target of the real time PCR is the ITS1 (The first internal transcribed spacer) (16). The real-time PCR assay (16) was performed in a 20 μl total volume containing Light cycler 480 probes master (Roche Diagnostic), 0.5 μM (each)primers AcanITS1F1 (5’TTCATGGATGGCGAACTGATAG-3’) and AcanITS1R1 (5’-GCGCCCATTGAAACATTATACTT-3’), 0.025 μM probe AcanITS1P1(5’FAM-ATCGCATATCTACTATACGCATGTGACACCTG-BHQ1-3’). and with 5 μl of DNA template. For prevention of carry over contamination we also use Uracyl DNA N-glycosylase (Roche diagnostics). The standard cycling conditions is: one cycle of 37°C for 5 min, one cycle 95°C for 5 min, 45 cycles of 95°C for 10 sec and 60°C for 50 sec and one cycle of cooling for 4°C for 10 sec. The positive control was *A*. *fulica* contaminated with *A*. *cantonensis* larvae. It was harvested from slugs via HCl (0.7%)–pepsin (0.5%) digestion (2 h, 37 ◦C), filtered, centrifuged and observed in optic microscopy to assess the presence of the larvae [18]. The negative control was water. Detection of inhibitors: To detect the presence of inhibitors of PCR and to verify the quality of the extraction we add 20 μl of Herpes simplex virus type 1 as internal control (AcroMétrix). The real time PCR of the control intern is realized separately. Conventional amplicons sequencing: Sequencing of conventional PCR products (105 pb) obtained from the nine positive patients was carried out using the ABI Prism BigDye Terminator Cycle Sequencing Ready Reaction kit version 3.1 (Applied Biosystems) with the two primers AcanITS1F1 and AcanITS1R1. The sequencing reaction was performed in a volume of 10 μL containing 1 μL PCR product template, 5.2 μL ddH_2_O, 2 μL sequencing buffer (5X), 1 μL oligonucleotide (4 μM) and 0.8 μL ABI Prism solution version 3.1. The sequencing program was performed as follows: 96°C 1 min followed by 30 cycles of 96°C 10 s, 50°C 5 s, 60°C 4 min. Sequence chromatograms for both strands were obtained using an automated sequence analyzer ABI3730XL (Applied Biosystems). Sequence analysis and alignment were performed using the software BioNumerics v 6.5 (Applied-Maths).

## Results

### PCR results

**Fig 1:**
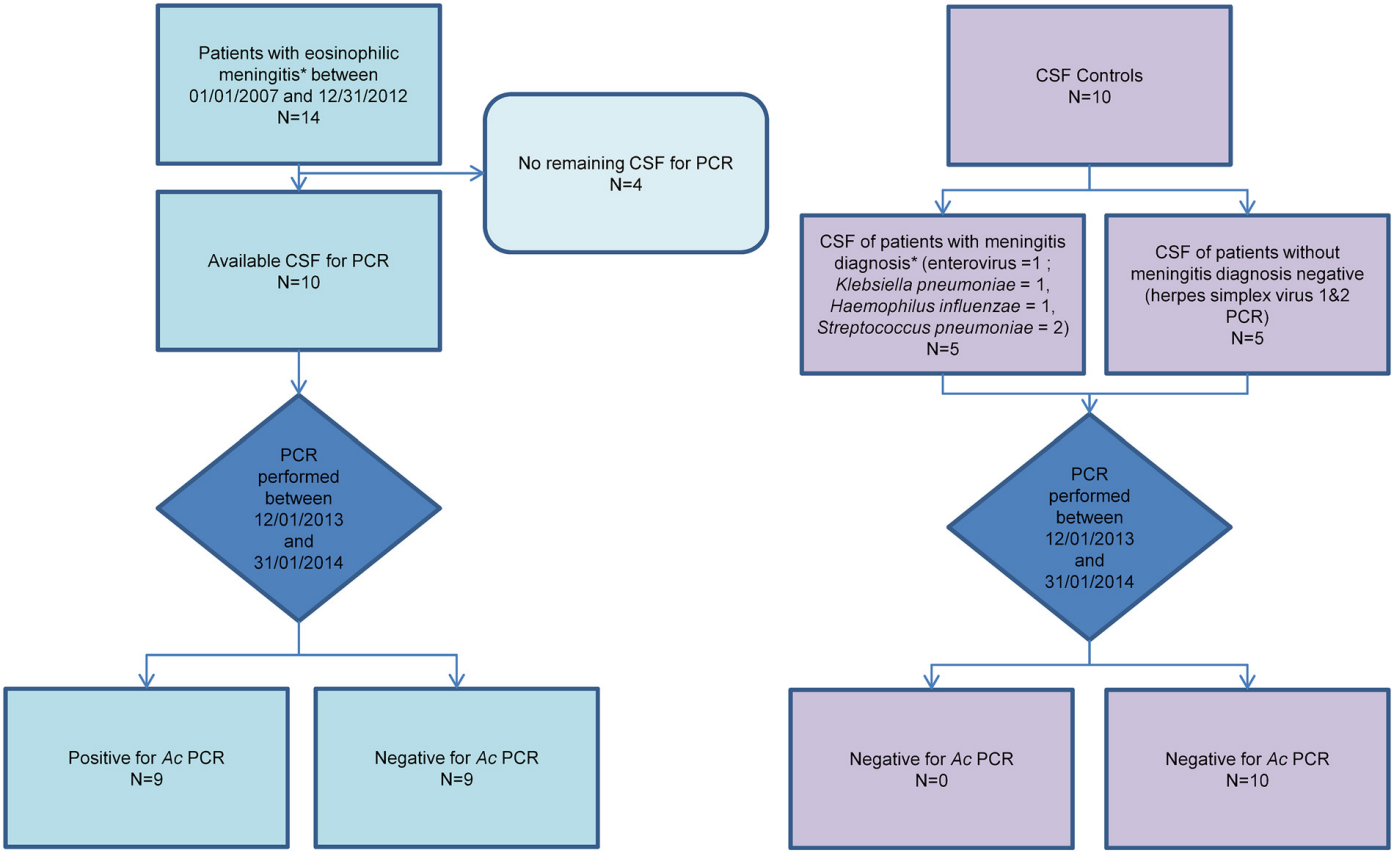
Flow chart of the study. *Ac*: *Angiostrongylus cantonensis*; CSF: Cerebrospinal fluid; PCR: Polymerase Chain Reaction. * Eosinophilic meningitis: ≥ 10 eosinophils /mm3 in CSF or ≥10% WBC). ** Meningitis: ≥ 10 WBC in CSF.

Fourteen patients with a diagnosis of EM were identified during the study period of 6 years from 2007 to 2013, e.g. an estimated incidence of 2.3 cases per year, and 1 case/year/100.000 inhabitants on the island. Ten were considered as presumptive and 4 probable according to the previous definition. Among them, 10 CSF samples were remained, and 9 of them (sensitivity 90%) were positive with real time PCR while 4/10 patients (40%) had a positive serodiagnosis in serum. PCR was negative for all CSF controls (Fig 1). Amplification curves of six patients positive by PCR and the positive control are represented on Fig 1.

**Fig 2:**
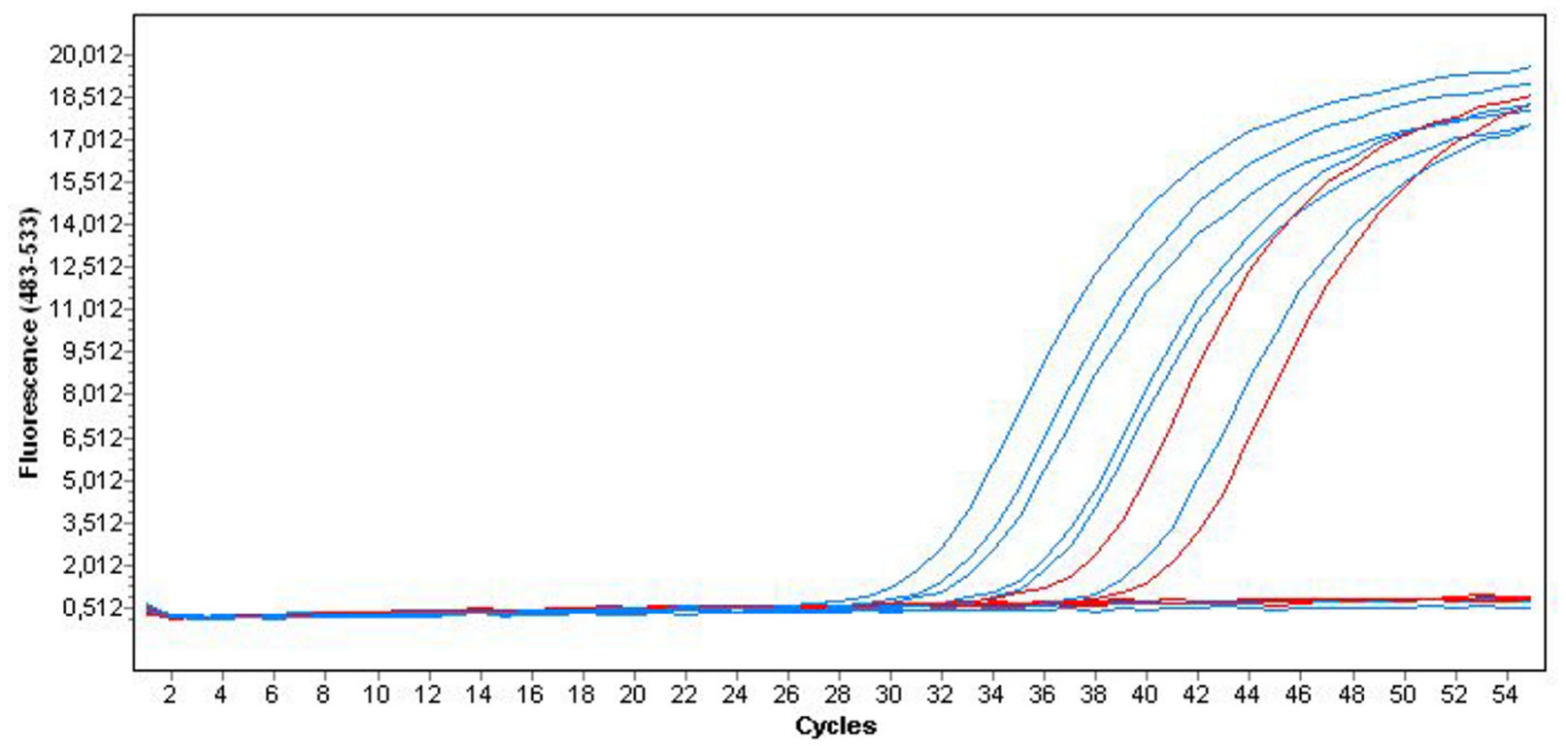
Amplification curves of six patients positive by Polymerase Chain Reaction and the positive control.

Sequencing of the nine individual conventional PCR amplicons (obtained from the 9 positive CSF) was done (Fig 2). The 105 pb obtained consensus sequence (TGCGCCCATTGAAACATTATACTTGGGTCATTAAGATTTCCTGTCAATCAGGTGTCACATGCGTATAGTAGATATGCGATGATACTATCAGTTCGCCATCCATGA) was strictly identical between the nine patients. BLASTn homology search against a non redundant nucleotide (nt) NCBI database for this consensus sequence showed 100% identity with *A*. *cantonensis* 18S ribosomal RNA gene, internal transcribed spacer 1, confirming the specificity of real-time PCR results.

### Clinical and biological characteristics

All 14 patients included in our series were children; thus 10 (71.4%) were under 12 month old and 13 (92.9%) were under 24 month old (Tables 1 and 2). Eleven (78.5%) were male, and 13 (92.9%) were born in Mayotte.

**Table 1:** Characteristics of the 14 children with nervous angiostrongyliasis and comparison with the 3 main available paediatric series in the literature.

Variable	Mayotte, 2015 (n = 14)	Taiwan, 1991 (n = 82) [16]	Thailand, 2013 (n = 19) [19]	Jamaica, 2014 (n = 6) [20]
**Anamnestic data**				
Male gender, N (%)	11 (78.5)	38 (46.3)	15 (78.9)	4 (66.6)
Season	Rainy season	Mostly summer, rainy	Summer	Winter
Exposure to intermediate host, N (%)	5/12 (41.7) Contact with *A*. *fulica*	71 (87) Contact with *A*. *fulica*	13 (68.4) Ingestion of freshwater snail	-
Incubation period, days		13.2 (mean)	22 (median)	
Age (year)*	0.8 (0.5–14)	~5 (0.8–14)	12 (4–14)	1.5 (1–8)
Age ≤ 24 month old	13 (92.9%)	26/82 (31.7)	-	-
Duration of symptoms before admission (days) *	7 (1–28)	-	-	-
**Clinical picture**				
Temperature (°C)*	38.1 (37.3–39.4)	-	-	-
Fever ≥ 38°C, N (%)	11 (78.5)	75 (91.5)	15 (78.9)	6 (100)
Digestive symptoms, N (%)	10 (71.4)	-	12 (63.2)	2 (33.3)
Vomiting, N (%)	3/10 (30.0)	51 (62.2)	-	-
Neurological symptoms, N (%)	13 (92.9)	-	-	5 (83.3)
Encephalitis signs, N (%)	8 (57.1)	25 (30.5)	0	1 (16.7)
Dysfunction of cranial nerves, N (%)	6 (42.9)	25 (30.5)	6 (31.6)	2 (33.3)
Seizure, N (%)	4 (28.6%)	-	-	0
Axial hypotonia, N (%)	3 (21.4%)	-	-	0
Abnormal cerebellar signs, N (%)	0 (0)		2 (10.5)	3 (50)
Hyperesthesia, N (%)	0 (0)	-	0	0
Headaches, N (%)	2 (14.3%)	-	19 (100)	2 (33.3)
Neck stiffness, N (%)	2 (14.3%)	-	13 (68.4)	1 (16.7)
**Brain imaging**				
Normal brain imaging	7/12 (58.3%)	9/16 (56.3)	-	-
Enlargement of brain ventricles	5/12 (41.7%)	3/16 (18.8)	-	-
Cerebral atrophy	5/12 (41.7%)	-	-	-
**Biological results**				
C-reactive protein (mg/L)*	13.5 (1–225)	-	-	-
Blood eosinophilia (/mm3)*	2400 (100–8400)	-	-	-
Blood eosinophilia (%)*	14.7 (1–31.8)	-	20 (5–48)	-
Eosinophilia ≥ 1000/mm^3^	13/14 (92.9)	-	-	-
Eosinophilia ≥ 10%	12/14 (85.7)	69 (84.1)	-	-
White cell count in CSF (/mm^3^)*	340 (54–1500)	-	637 (87–2610)	244 (80–640)
White cell count ≥ 100/mm^3^	11/14 (78.6)	76 (92.6)	-	
Eosinophilia in CSF (/mm^3^)*	194 (3–690)	-	-	14 (11–20)
Eosinophilia in CSF (%)*	48 (5–76)	62.2 (51–90)	58 (31–95)	-
CSF glucose (mmol/L)*	2.3 (1.1–3.5)	-	3.8 (1.9–13.3)	-
Protein level in CSF (g/L)*	0.75 (0.2–1.2)	-	0.45 (0.26–1.14)	-
Protein level > 0.45 g/L	10/14 (71.4)	41/67 (61.2%)	-	-
*A*. *cantonensis* positive serodiagnosis	4/10 (40%)	34 (41.5)	-	-
Real time PCR in CSF	9/10 (90%)	NR	-	-
Worm recovery in CSF (%)	0	25 (30.5)	0	2
**Outcome**				
One month mortality rate	2/14 (14.3%)	4/82 (4.9)	0 (0)	0 (0)
One month neurological sequelae	3/12 (25%)	-	-	2/6 (3.6)
One year mortality	5/14 (35.7%)	-	-	0 (0)
One year neurological sequelae	3/7 (42.8%)	-	-	2/6 (3.6)

* Median, range

CSF: Cerebro-spinal fluid; RT PCR: Real-Time Polymerase Chain reaction

**Table 2:** Characteristics of the 14 patients with *Angiostrongylus cantonensis* infection.

Case	sexe	Age (month)	Reported contact with mollusk	Duration of symptoms before admission (days)	neurological symptoms	Digestive symptoms	Eosinophil count in blood (/mm3 and %)	CRP (mg/L)	Eosinophil count in CSF (/mm3 and %)	Glucvose level in CSF	Protein level in CSF	Angiostr ilus serodiagnosisIFI Acute/Convalescent phase	Ac RT PCR in CSF	Brain CT-scan	Brain MRI	Treatment	Status 1 month after admission	Status 1 year after admission	Neurocognitive sequelae
1	M	9	No	13	Axial hypotonia/Seizure	-	5300 (33,3)	152	690 (46)	1,7	1,2	*-*/**Positive**	-	Normal	-	Albendazole/Ivermectin	Alive	Alive	None
2	F	12	S-il	7	Encephalitis/Dysfunction of cranial nerves	Yes, unspecified	2600 (22,6)	17	26 (48)	1,1	0,7	*cf*	positive (34,6)	Ventricular dilatation / Cerebral Atrophy	-	Albendazole/Flubendazole/ivermectine and corticosteroids	Deceased	Deceased	-
3	M	7	No	15	Axial hypotonia/Headaches	Yes, unspecified	3200 (15,8)	4,3	144 (40)	2,4	0,8	*Negative*/-	positive (33,8)	Normal	-	Ivermectin/Mebendazole and corticosteroids	Alive	Alive	Unknown
4	M	8	No	7	Encephalitis/Dysfunction of cranial nerves	No	3000 (14)	10	3 (5)	3,1	0,3	*Negative*/**Positive**	positive (31,8)	Ventricular dilatation / Cerebral Atrophy	-	Ivermectin/Mebendazole and corticosteroids	Deceased	Deceased	-
5	M	9	No	7	Encephalitis Dysfunction of cranial nerves	Anorexia	100 (1)	44,5	230 (72)	2,1	0,8	*Negative*/**Positive**	positive (29,5)	Ventricular dilatation / Cerebral Atrophy	-	Albendazole and corticosteroids	Alive	Deceased	-
6	M	9	S-il	7	Encephalitis/Seizure	Anorexia	2300 (13)	2	100 (40)	2,8	0,9	*Negative*	-	Ventricular dilatation / Cerebral Atrophy	-	Acetazolamid/Ivermectin/Mebendazole and corticosteroids	Alive	Alive	Psychomotor retardation/epilepsy
7	F	10	Slug	1	Axial hypotonia/intracranial hypertension	Yes, unspecified	1500 (15,4)	5,5	207 (66)	3,4	0,2	*Negative*/None done	positive (38,7)	Normal	-	Ivermectin/Mebendazole	Alive	Alive	Psychomotor retardation
8	F	11	No	15	Encephalitis Dysfunction of cranial nerves/Seizure	Anorexia/abdomi-l pain	2000 (12,1)	73	38 (70)	1,4	0,5	*Negative*/-	-	Ventricular dilatation Cerebral Atrophie/hydrocéphalie externe, hyperhémie méningée	-	Ivermectin and corticosteroids	Alive	Deceased	-
9	M	10	S-il	1	Seizure	No	2000 (16)	6,2	210 (56%)	3,5	0,4	-	negative	Normal	-	Corticosteroids	Alive	Alive	None
10	M	24	S-il	15	Headaches	-useas and vomits	2500 (13.2)	<5	180 (45)	2,2	0.73	Positive/-	positive (35,9)	Normal	Small scattered hemorrhagic foci (subarachnoid hemorrhage sequellae)	Albendazole and corticosteroids	Alive	Alive	None
11	M	168	-	28	None	Vomits/Abdomi-l pain	5300 (31.2)	<5	300 (40)	2,7	0.80	negative	positive (30,5)	-	-	Albendazole	Alive	Alive	Psychomotor retardation
12	M	10	No	1	Hemiparesis/Meningeal syndrome	No	8400 (31.8)	225	494 (76)	2,1	0.77	negative	positive (31,9)	Normal	-	Albendazole/Ivermectin	Alive	Alive	None
13	M	21	No	5	Dysfunction of cranial nerves/meningeal syndrome	Vomits/Abdomi-l pain	2000 (17.3)	18	25 (12)	2,8	0.28	-	positive (32,8)	-	Normal	Albendazole and corticosteroids	Alive	Alive	None
14	M	13	-	-	Dysfunction of cranial nerves	Diarrheoa	1000 (8)	46,5	504 (56)	1,7	1	-	-	-	-	-	Alive	Deceased	-

CSF: Cerebro-spinal fluid; RT PCR: Real-Time Polymerase Chain reaction, CT-scan: computerized tomography scan; CRP: C-Reactive Protein

At admission, 11/14 children (71.4%) had fever, and 9 digestive symptoms (abdominal pain, vomiting and/or diarrhea). Besides the 14-year-old previously disabled, all children presented with acute neurological symptoms (13/14): encephalitis signs (n = 9), dysfunction of cranial nerves (n = 6), seizures (n = 4), axial hypotonia (n = 3), neck stiffness (n = 2), headaches (n = 2). Brain imaging was performed in 12 of them (10 CT-scan, 1 MRI, 1 CT-scan + MRI): seven had a normal brain imaging and 6 presented abnormalities with cerebral atrophy and abnormal enlargement of cerebral ventricles for 5 of them.

Blood eosinophilia was constant with a median of 2400/mm^3^, with an eosinophilia level rising above 1000/mm^3^ in 13 (92.9%) patients (Table 1). Median eosinophilia in CSF was 194 (48%), with a range of 35–690/mm^3^ (12–76%). Other CSF analysis found a moderate protein level elevation in CSF and normal or low glucose level.

A contact with mollusks was reported by the parents in 5/11 cases (41.7%), 4 with AGS, and 1 with a non identified slug.

### Follow-up and variables associated with mortality

The lethality rate was high and accounted at 14.3% (2/14) at 1 month after admission and 35.7% (5/14) at 1 year of follow-up. Among the 9 children still alive after one year, the neurological, state couldn’t be evaluated in two of them, and 3/7 (42.8%) presented neurological sequelae. No difference for lethality was evidenced for age, gender, blood eosinophilia, eosinophilia and protein levels in CSF and treatment used between alive and deceased children at one year (Table 3).

**Table 3:** Comparison of dead and alive children 1 year after *A*. *cantonensis* infection.

Variable	Alive (n = 9)	Deceased (n = 5)	p**
Male gender	8 (88.9%)	3 (60%)	0.2
Age (month)*	10 (9–168)	11 (8–13)	0.6
Age ≤ 12 month old	6 (66.7%)	3 (60%)	0.8
Symptoms ≥ 7 days before admission	5 (55.6%)	4 (80%)	0.1
Fever (T°≥38°C)	5/8 (62.5%)	3/4 (75%)	1
Digestive symptoms	5 (62.%)	3 (60%)	0.9
Seizure	3 (33.3%)	1 (20%)	0.6
Encephalitis signs	5 (55.6%)	4 (80%)	0.3
Dysfunction of cranial nerves¤	1 (16.7%)	5 (100%)	0.001
Abnormal brain imaging¤	2 (22.2%)	4 (80%)	0.04
C-reactive protein (mg/L)*	5.9 (1–225)	44.5 (17–73)	0.3
Blood eosinophilia (/mm3)*	2400 (1500–8400)	2000 (100–3000)	0.3
Blood eosinophilia ≥ 2500/mm3	4 (44.4%)	2 (40%)	1
White cell count in CSF (/mm3)*	388 (213–1500)	54 (54–320)	0.16
White cell count in CSF > 300/mm3	7 (77.8%)	2(40%)	0.16
Red cell count in CSF > 50/mm3	3 (33.3%)	2 (40)	0.8
Eosinophilia in CSF >200/mm3	5 (55.6%)	2 (40%)	0.6
Eosinophilia in CSF >50%	3 (33.3%)	3 (60%)	0.3
CSF glucose level < 2 mmol/L¤	2 (22.2%)	4 (80%)	0.04
Protein level in CSF > 0.6 g/L	6 (66.7%)	3 (60%)	0.8
Treatment with albendazole	5 (55.6%)	2/4 (50%)	0.8
Treatment with ivermectin	4 (57.1%)	3/4 (75%)	0.3
Treatment with corticosteroids	5 (55.6%)	4/4 (100%)	0.1

* Median, minimum, maximum

** p calculated with Fisher’s exact test for categorical variables and with Mann-Whitney Test for continual variables

^¤^ Variables with significant differences between the two groups

CSF: Cerebro-spinal fluid;

There was a significative association between dysfunction of cranial nerves (p = 0.001), abnormal brain imaging (p = 0.04) and a CSF glucose level inferior to 2 mmol/L (p = 0.04) and the mortality at one year. A history of clinical manifestations over 7 days before admission, and eosinophilia superior to 50% of CSF cell count tended to be more frequent in deceased children but not significantly.

### Identification of a source of transmission

**Fig 3:**
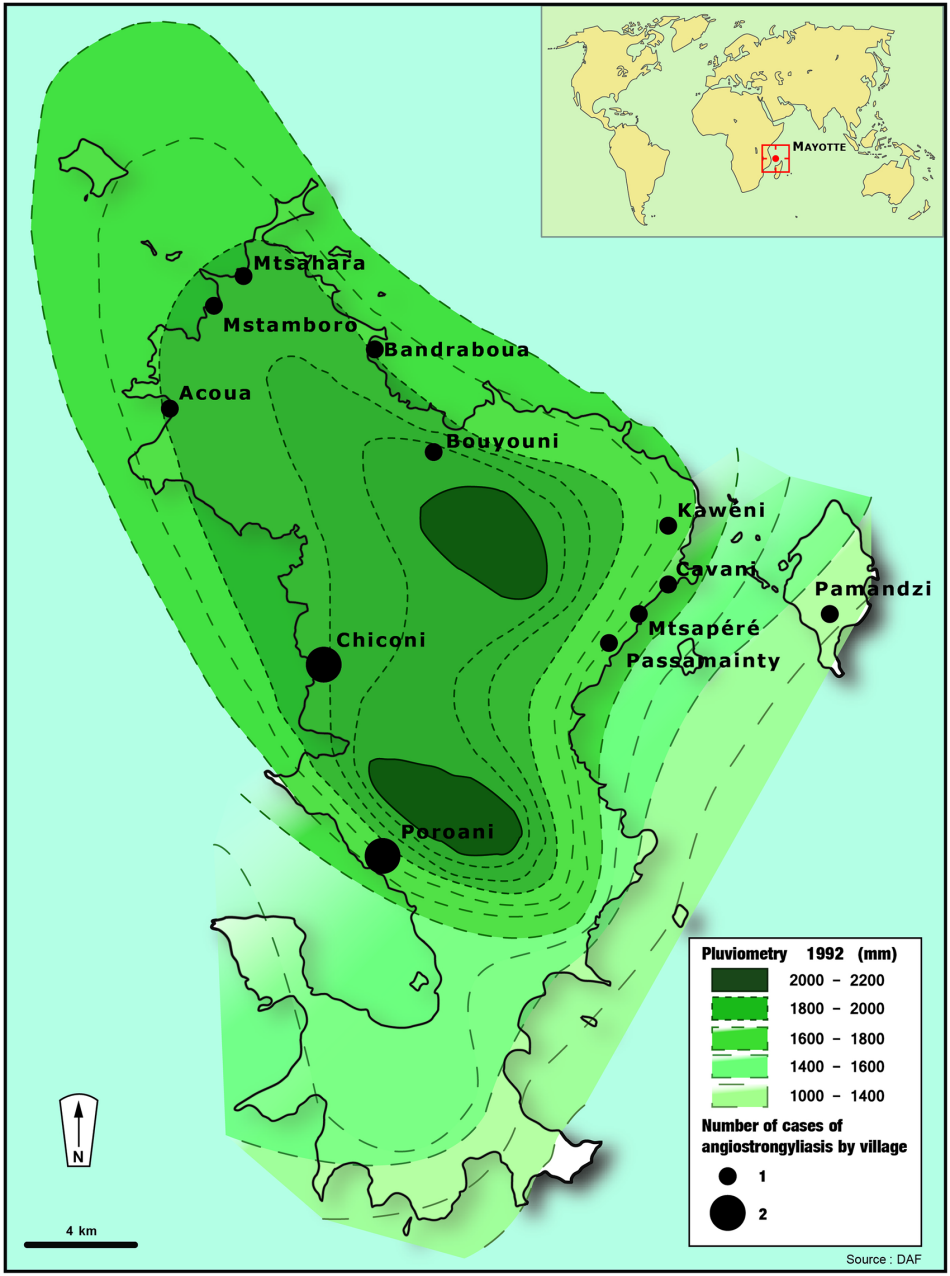
Map of the island of Mayotte representing the number and location of occurrence of the cases of angiostrongyliasis and the pluviometry curves. Figure 3 was created using Illustrator CS5 (Adobe Systems, Inc.).

**Fig 4:**
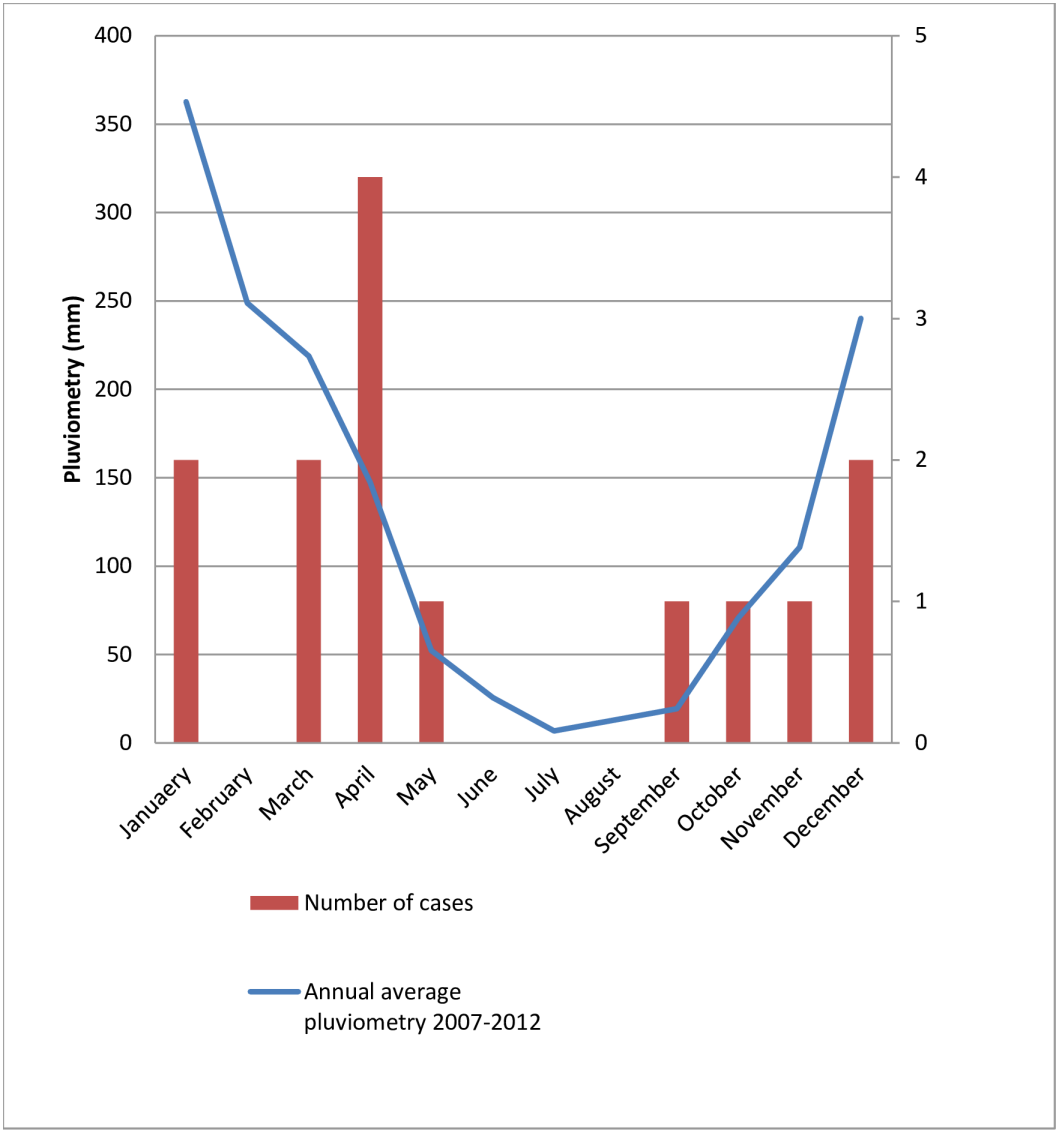
Monthly repartition of the cases and average pluviometry.

Spatial localization highlighted that the places of residence of all cases were located on the northern part of the island. Of note, this area is the rainiest part of Mayotte (Fig 3). Date of occurrence of the cases and rainfall precipitations curves were superimposed on the same sketch arguing for a putative association between rainfall and transmission. Consistently, most cases were evidenced during the rainy season (Fig 4). Among all the snails collected in the different areas of the island, 6/64 (9.4%) (from 0 to 2/8 samples, depending on the village) were positive by *A*. *cantonensis* PCR for, with a likely high parasitic load (Table 4).

**Table 4:** Number of *Achatina fulica* (African Giant Snail) positive to *Angiostrongylus cantonensis* with real time PCR and number of collected snails in the place of residence of every sick child.

Village	Real time PCR	CT
Cavani	0/5	-
Kaweni	0/5	-
Pamandzi	0/5	-
Acoua	0/5	-
Passamainty	0/5	-
Poroani	0/5	-
Mtsamboro	2/8	22,2/39,4
Mtsapéré	1/11	21,0/
Chiconi	1/5	24,9/
Bandraboua	1/5	26,4/
Bouyouni	1/5	24,8/
**TOTAL**	**6/64 = 9,4%**	**-**

Real time PCR: Real-Time Polymerase Chain Reaction; CT: threshold cycle

## Discussion

### Characteristics of HA endemic of Mayotte

On Mayotte, a small island located in the South-West part of Indian Ocean, HA cases occur mainly in very young children: our study reports 14 cases, among which 13 were children beneath 2 years old. The remaining patient was a 14-year-old disabled child suffering of sequelae of bacterial meningitis in the childhood. He was regularly playing with snails, lying on the grass all the day. Whether the incidence is very low (2.3 cases per year, e.g. approximatively, 1 case/100,000 inhabitants/year), the rate of morbidity and lethality is very high with a 1-year mortality of 38.5% and incapacitation rate of 37.5% among the remaining 8 children. Some case reports have already been published on *A*. *cantonensis* infection in Mayotte [11, 12, 21]. Most of these reported cases presented severe clinical pictures in infants, although some cases in adults have been described [13]. Thus, human angiostrongyliasis seems to have very high morbidity and mortality rates, never described in the medical literature, according to our knowledge. Such a severity in children has never been reported before. It is considered that most cases of human angiostrongyliasis are generally mild and self-limiting, even if death can occur in severe cases [3]. Indeed, the first pediatric case-series performed in Taiwan and published in 1991 reported a mortality rate of 4.9% among 82 children [16]. Of note, children included in the latter study were older than those for our study: 58.5% < 6 yo and 80% < 9 yo. This main issue might account for the critical issue evidenced in our series and the age of the patients has already been evocated as a possible explanation [22]. A series of 19 cases of eosinophilic meningitis due to A. cantonensis from Thailand was published in 2013 [19]. No death was reported in this publication. In this series fever and digestive signs were as frequent as in ours, but headache (14.9 vs. 100%) and neck stiffness was more frequent. On the contrary, severe clinical presentations was wore frequent in our child patients than in the Thailand’s ones: dysfunction of cranial nerves (42.9 vs. 31.6%), encephalitis signs (57.1 vs. 0%). In this series, the main route of transmission was likely the consumption of raw freshwater snails while the children in Mayotte are contaminated to the contact of *A*. *fulica*. In another publication from Thailand comparing encephalitis vs. meningitis cases (14 vs. 80 cases respectively), death was strongly linked to encephalitis (79 vs. 0%) [23]. In another Taiwanese study, with 37 Taiwanese patients diagnosed over an 18-year period (two were children), neurological sequelae developed in only one 2-year-old child. Authors evocated that a higher worm load is relative to body size would explain the severity of the disease [24]. In our series, encephalitis signs were frequent and may explain the high mortality. The main hypothesis to explain the frequency of the encephalitis may be the high parasitic load in the snails and also the low age of the children, with a quicker progression to central nervous system infection.

Recently a study was published reporting few severe cases in children in Jamaica [20]. Nevertheless, in our study, there was no difference in terms of age between alive and deceased children, even if they were all very young. Impact for age was found according to outcome.

Despite the small size of our sample, some variables were associated with a higher risk of 1-year mortality such as abnormal brain imaging was associated, dysfunction of cranial nerves, although it was considered as an usual symptom in previous large series [22] and a low CSF glucose level. This finding might reflect a higher parasitic load in the CSF and an stronger immunological response, both biological factors linked to severity of diseases.

Our study evidenced for the first time that Mayotte’s *A*. *fulica* was carrier of *A*. *cantonensis*. Nevertheless, the rate of carriage was quite low (9.4%) in comparison to other places such as Hawaii (72.6%), São Gonçalo, a metropolitan area of Rio de Janeiro, Brazil (78.7%), Miami, Florida (36%), but closer to China’s investigations (13.4%) [25–28]. Nevertheless, parasitic load reflected by real PCR results were very high, so both could explain at the same time the low incidence of the disease in our island, but the severity of the clinical picture. At last, no treatment either corticosteroid or antiparasitic therapy demonstrated a benefit on the outcome of the disease, especially in case of encephalitis and comatose [14, 29]. In the present study, deceased children received more often corticosteroids than survivors. This observation might account for intensive suppletive cares related to severe presentation.

### Route of transmission

The main hypothesis for the contamination with *A*. *cantonensis* in Mayotte is the contact of the children with *A*. *fulica*. Thus, very few cases have been described in adults, and the route of transmission was not known [13]. Thus, the route of transmission of human angiostrongyliasis remains unclear in adults in Mayotte because people from the whole Comoros archipelago don’t eat snails, slugs, nor shells and any food is very well cooked. The infections could be linked to the consumption of raw vegetables contaminated with infected gastropod’s slime. On the contrary, the route of transmission is clearer in infants who are directly in contact with the host omnipresent in the environment on the island, and they lick snails and slugs or their hands contaminated with it. This also explains why cases occur during the rainy season (Fig 4) and the wettest part of the island (Fig 3), as *Achatina fulica* aestivates during dry season. Thus, the transmission cycle is completely different in Thailand where it mainly affects adults, by consuming raw foods, and where transmission is throughout the year and is more similar to transmission cycle as described in Taiwan [16]. Indeed, almost 10% of the local AGS were proven to be carriers of the parasite in Mayotte using the real time PCR. *A*. *fulica* is recognized to be one of the main vector of the disease in many settings [3] and it has already been evidenced in other islands of the Indian Ocean such as La Réunion and Madagascar [7]. We were able to find a close contact between children and snails in only 4/11 cases, nonetheless, reports might be limited by declarative or memory bias, due to the retrospective settings of the study. Furthermore, one mother reported that her son played with an unidentified slug rather than with snails, which might evoke that AGS is not the only vector on the Island. Many other mollusks host have been described to be infected by the parasite, like in Brazil [30]. Direct contact with *A*. *fulica*, as a route of transmission has been barely reported among adult patients [3, 14]. Distinctly, Hwang et al, in the pediatric study published in 1991 reported an history of contact between *A*. *fulica* and the patients in more than 80% of the cases [16]. This mode of transmission may partly explain the specific epidemiological features of Mayotte, with mainly pediatric cases compared to the great endemic area, such as Southeastern Asian countries, where adults are the most infected. In those countries, the main route of transmission is the consumption by adults of the raw or undercooked host, such as shellfish or snails [14]. The ingestion of vegetables contaminated with snail slime is also often reported, such as during a cluster in travelers returning from Jamaica [6, 31]. Yet, in the Comoros archipelago, giant African snails are not commonly eaten, unlike in some sub-Saharan continental African countries, such as Nigeria [32], as well as fresh water shellfish. Adult cases have been rarely reported in Mayotte: only three before our study [13], but the source of transmission was not clearly identified for these patients to the best of our knowledge.

As previously stated, it would be very difficult to propose environmental preventive measures. Indeed, *A*. *fulica*, initially native to East Africa [33], is widely spread on the island [34], and a minority (<10%) seems to be infected. *A*. *fulica* is listed as one of the top 100 worldwide introduced invasive species (100 of the Worst Invasive Species. Global Invasive Species Database. URL: http://www.issg.org/database/species/search.asp?st=100ss), so that may encourage people to try to exterminate it, as people consider it as a recent invasive species on Mayotte. Nevertheless, archaeological studies estimated that this snail would be present on the island since at least the 8^th^ century of our era [35]. Thus, the most important preventive measures are individual and consist in advertise the population to avoid the contact with snails and slugs, and not to let their children play with them, especially during the rainy seasons. Indeed, we found that most cases occur during this period, particularly suitable for *A*. *fulica*, and in the wettest part of the island. It has been previously demonstrated that AGS are rare during the dry season as they aestivate because of the hot dry weather and they bury themselves in the soil or hide beneath stones in order to avoid exposure to direct solar radiation [36].

### *Angiostrongylus cantonensis* real time PCR

*A*. *cantonensis* real time PCR was an essential tool to complete the study. It demonstrated a very sensitivity (90%) for the retrospective definite diagnosis of HA, and with a specificity of 100%. On the contrary, the sensitivity of serodiagnosis was very low (60%), and helminths serodiagnosis, especially for *A*. *cantonensis*, induce many false positive due to cross reaction with other helminths, such as *Strongyloides stercoralis*. Furthermore, no laboratory performs this test it in France anymore. There are few publications about the use of real time PCR for human cases diagnosis. Since the first publication of the biological method in 2010 [17], it has been mainly used for the identification of the vector, especially in Hawaii, USA and Brazil [25, 37, 38]. Some publications reported the use of real time PCR for the diagnosis of human angiostrongyliasis, and not always successfully, in Hawaii and in Brazil [39, 40]. We present here the most important positive case series, and demonstrate the great interest of this tool for the confirmatory diagnosis. This skill is especially of interest if we acknowledge that there are yet reliable PCR testing for the diagnosis of neurological helminthiasis, such as cysticercosis, toxocariasis, schistosomiasis, paragonimiasis, gnathostomiasis and baylisascariasis, and which are the differential diagnosis of nervous HA of eosinophilic meningitis [41, 42]. Nevertheless, the ideal control group for PCR would have been CSF of gnathostomiasis, which is the main differential diagnosis of HA. This control would have given very high diagnostic properties. Unfortunately we were not able to find this control, because, gnathostomiasis has never been reported on Mayotte, and other causes of eosinophilic meningitis are scarce. Thus, the high diagnostic properties of PCR in this study may be due to inappropriate control group.

In conclusion, in Mayotte, HA mainly affects infant under 2 years and is a highly life-threatening disease. Real time- PCR seems to be a powerful and promising tool in the definitive diagnosis of HA. Whether eradication of the vector is illusory, the population should be aware of the risk of direct contact with the African giant snail, and try to educate the children to avoid it. In endemic areas, every physician may suspect the disease in front of any febrile neurological pictures, and a lumbar puncture should be performed as soon as possible to realize the PCR on the CSF as well as brain imaging.

## Supporting Information

S1 Checklist STROBE checklist. (DOC)

S2 Checklist STARD checklist. (DOCX)
